# GIS-supported epidemiological analysis on canine *Angiostrongylus vasorum* and *Crenosoma vulpis* infections in Germany

**DOI:** 10.1186/s13071-017-2054-3

**Published:** 2017-02-28

**Authors:** Pavlo Maksimov, Carlos Hermosilla, Anja Taubert, Christoph Staubach, Carola Sauter-Louis, Franz J. Conraths, Majda Globokar Vrhovec, Nikola Pantchev

**Affiliations:** 1 Friedrich-Loeffler-Institut, Federal Research Institute for Animal Health, Institute of Epidemiology, Greifswald-Insel Riems, Germany; 2 Institute of Parasitology, Justus Liebig University Giessen, Giessen, Germany; 3 IDEXX Laboratories, Ludwigsburg, Germany

**Keywords:** *Angiostrongylus vasorum*, *Crenosoma vulpis*, Epidemiology, Risk factors, Dogs

## Abstract

**Background:**

*Angiostrongylus vasorum* infections are the cause of severe cardiopulmonary diseases in dogs. In the past, canine angiostrongylosis has largely been neglected in Europe, although some recent studies indicated an expansion of historically known endemic areas, a phenomenon that might also apply to *Crenosoma vulpis*. The aim of the present study was to analyse temporal and spatial trends of canine *A. vasorum* and *C. vulpis* infections and to perform GIS-supported risk factor analysis to evaluate the role of landscape, age and seasonality in the life-cycle of these nematodes.

**Methods:**

A total of 12,682 faecal samples from German dogs (collected in 2003–2015) with clinical suspicion for lungworm infection were examined for the presence of *A. vasorum* and *C. vulpis* larvae by the Baermann funnel technique and respective epidemiological data (location and age of the sampled dogs, date of sampling) were subjected to GIS-supported risk factor analysis.

**Results:**

Overall, *A. vasorum* and *C. vulpis* larvae were detected in 288 (2.3%) and 285 (2.2%) faecal samples, respectively. In general, both lungworm infections were found to be widely spread in Germany. GIS-supported analyses demonstrate spatial differences in the occurrence of canine *A. vasorum* and *C. vulpis* infections in Germany*.* also, risk factor analyses revealed an overlap but also diverging risk and protective factors for *A. vasorum* and *C. vulpis* infections. The current data also indicate a significant increase of *A. vasorum* and *C. vulpis* prevalences from 2003 to 2015 and from 2008 until 2015, respectively, and a potential spread of *A. vasorum* endemic areas to the northeastern part of Germany.

**Conclusions:**

The results of the present study show an insight into the epidemiological situation of lungworm infections (*A. vasorum* and *C. vulpis*) of the past 13 years in Germany. The data clearly demonstrate an increase of diagnosed *A. vasorum* prevalence in the tested dog population between 2003 and 2015 as well as spatial differences in the occurrence of diagnosed *A. vasorum* and *C. vulpis* infections of dogs in Germany*.* Risk factor analyses suggest possible differences in the biology of these parasites, presumably at the intermediate host level.

**Electronic supplementary material:**

The online version of this article (doi:10.1186/s13071-017-2054-3) contains supplementary material, which is available to authorized users.

## Background

In recent years, canine *Angiostrongylus vasorum* infections received attention due to their spread beyond the borders of known endemic areas and the association of the infection with coagulopathies, neurological disorders, pulmonary hypertension, systemic disease and sometimes death in dogs as definitive hosts [1–4]. While *A. vasorum* adults reside in the *arteria pulmonalis* and the right heart of the hosts [5], *Crenosoma vulpis* persists in the bronchi, bronchioles and occasionally in the trachea of canids as final hosts and may induce dry coughing and bronchitis [6]. *Crenosoma vulpis* infections are rarely fatal, but canine crenosomosis is also recognised as an important cause of chronic respiratory disease in dogs [3, 7–11]. While *C. vulpis* is known to be endemic and evenly spread in areas of North America and Europe with a temperate climate, *A. vasorum* infections typically show “patchy” distribution patterns with stable endemic foci being surrounded by areas of low or absent parasite occurrence [2, 3, 11–16]. In recent years, a spread beyond these traditional endemic areas has been reported [2, 4, 17].

Some reports deal with factors influencing the biology and spread of *A. vasorum* infections. As such, an altitude of 700 m over sea level was reported as a limiting factor for *A. vasorum* transmission [18]. Morgan et al. [14], as well as Barutzki et al. [11], demonstrated that younger dogs are at higher risk of *A. vasorum* infection. The seasons also influence the infection dynamics of *A. vasorum* as a greater number of cases was found in the first months of the calendar year [3, 14]. Furthermore, a deworming history had protective effects against *A. vasorum* infections [14].

Apart from these data, the epidemiology of canine angiostrongylosis is still poorly understood. Also, scarce information is available on potential risk factors for *C. vulpis* infection [3]. There is little information on the possible impact of landscape or land-use on *A. vasorum* and *C. vulpis* prevalences, which may affect the epidemiological situation in intermediate and final hosts. Thus, the purpose of this study was to perform GIS-supported epidemiological analyses by using digital landscape data in Germany. Besides landscapes, the influence of the season and age was analysed as risk/protective factors. Furthermore, we intended to address the question, whether the prevalences of both lungworms truly in/decreased during the period of investigation (2003–2015) in the study area in Germany.

## Methods

### Sample collection

Faecal samples of 12,682 dogs from Germany were submitted by veterinary surgeons to a private veterinary diagnostic laboratory (IDEXX Laboratories, Ludwigsburg, Germany) between 2003 and 2015 and examined for lungworm larvae using the Baermann funnel technique according to Taubert et al. [3, 19]. The following epidemiological data were collected: sampling locations (postcode), the age of the sampled dogs and sample submission dates. No information was available about the attitude and clinical symptoms of the sampled dogs. It is worth noting that the veterinary surgeons actively ordered a test for the detection of lungworm larvae on the official submission form of the laboratory, although it was also possible to order the specific testing for *A. vasorum* infection. Consequently, it can be assumed that most dogs were presented with a clinical suspicion for lungworm infection. Most submissions consisted of a single faecal sample.

*Crenosoma vulpis* and *A. vasorum* first stage larvae were identified based on morphological findings at the larval tail according to Georgi et al. [20, 21]: the tapered tip of the tail of *A. vasorum* first larvae has a kink with a dorsal spine, whilst that of *C. vulpis* tapers smoothly, lacking any kink, undulation or spine [3].

### Geographic information system (GIS) analyses

Data were compiled in an Excel spreadsheet (Microsoft® Office 2010) and the R software package [22]. For risk factor analyses, independent variables were included, which may be positively or negatively associated with *A. vasorum* and *C. vulpis* positivity in Baermann funnel tests. Bivariate variables containing *A. vasorum* and *C. vulpis* positivity status (“1” as positive and “0” as negative) were used as dependent variables. The following independent variables were used: (i) age of the sampled dog in months, which ranged from 14 days to 264 months (22 years). The median of the age was 48 months (4 years); (ii) seasonality of *A. vasorum* and *C. vulpis* infections, here the overall monthly prevalence of the corresponding lungworm was applied as derived from the sample submission date; and (iii) the proportion of land-use classes per postcode (bodies of water, traffic, agricultural field, other agriculture, woody plant area, moorland, broadleaf forest, softwood forest, mixed forest, bog, leisure/sport park area, housing area) as derived from a digital landscape model (DLM) of the national land survey vector database (1:25,000; overall precision ± 3 m) ATKIS (Amtliches topographisch-kartographisches Informationssystem, Bundesamt für Kartographie und Geodäsie, Frankfurt, Germany; http://www.geodatenzentrum.de).

Land-use data were referred to postal code areas as the common spatial unit and maps plotted using ArcGIS software (version 10.2.2; ESRI, Redlands, CA, USA). The measurement unit in the GIS was one square meter using a Transversal Mercator Projection with Bessel Ellipsoid. An overlay analysis was performed by intersecting the postal code areas with the ATKIS data as described by Kuerpick et al. [23] to determine land-use composition.

All postcodes with the first two identical numbers were merged into a postcode district to simplify the spatial presentation of *A. vasorum* and *C. vulpis* prevalence data.

### Statistical analyses

For statistical analyses and graphical presentations, the R software environment for statistical computing and graphics was used [22]. The R libraries Splines, glmmML, lme4, epicalc, epiR, classInt, ggplot2, fmsb, multcomp, spdep, maptools and aod were applied. Univariable analysis was performed by logistic regression as described elsewhere [23, 24]. Briefly, variables described in the previous section were tested against the Baermann funnel data of both lungworms using the R package “*stats*”.

We also performed a multivariate analysis, where variables with *P*-values ≤ 0.2 in the bivariate analysis were included in the full model. The models were constructed in such a way that they always contained the variables potentially related to the life-cycle of *A. vasorum* and *C. vulpis,* i.e. age and seasonality. Akaike’s Information Criterion (AIC) was calculated for each model and used as the selection parameter for the final model [23]. Stepwise model building modified the variables of the full logistic regression model with automated forward and backwards selection of variables using the R package “*MASS*” to identify the most parsimonious model providing the lowest AIC value.

To assess the proportion of variation of *A. vasorum* and *C. vulpis* positivity in Baermann funnel testing explained by the models, pseudo-*R* ^*2*^ values were calculated with R package “*rms*”. The predictive performance of the models was characterised by receiver operating characteristics (ROC) analysis and determination of AUC (Area Under Curve) values [23, 25] using the R package “*Presence/Absence*”.

As several samples may have been exposed to similar landscape types, a general linear mixed model with the postal codes at a random intercept level was used to consider the possible influence of correlation of independent variables.

## Results

### Prevalence per postcode district of canine *A. vasorum* and *C. vulpis* infections in Germany for 2003–2015

**Fig. 1:**
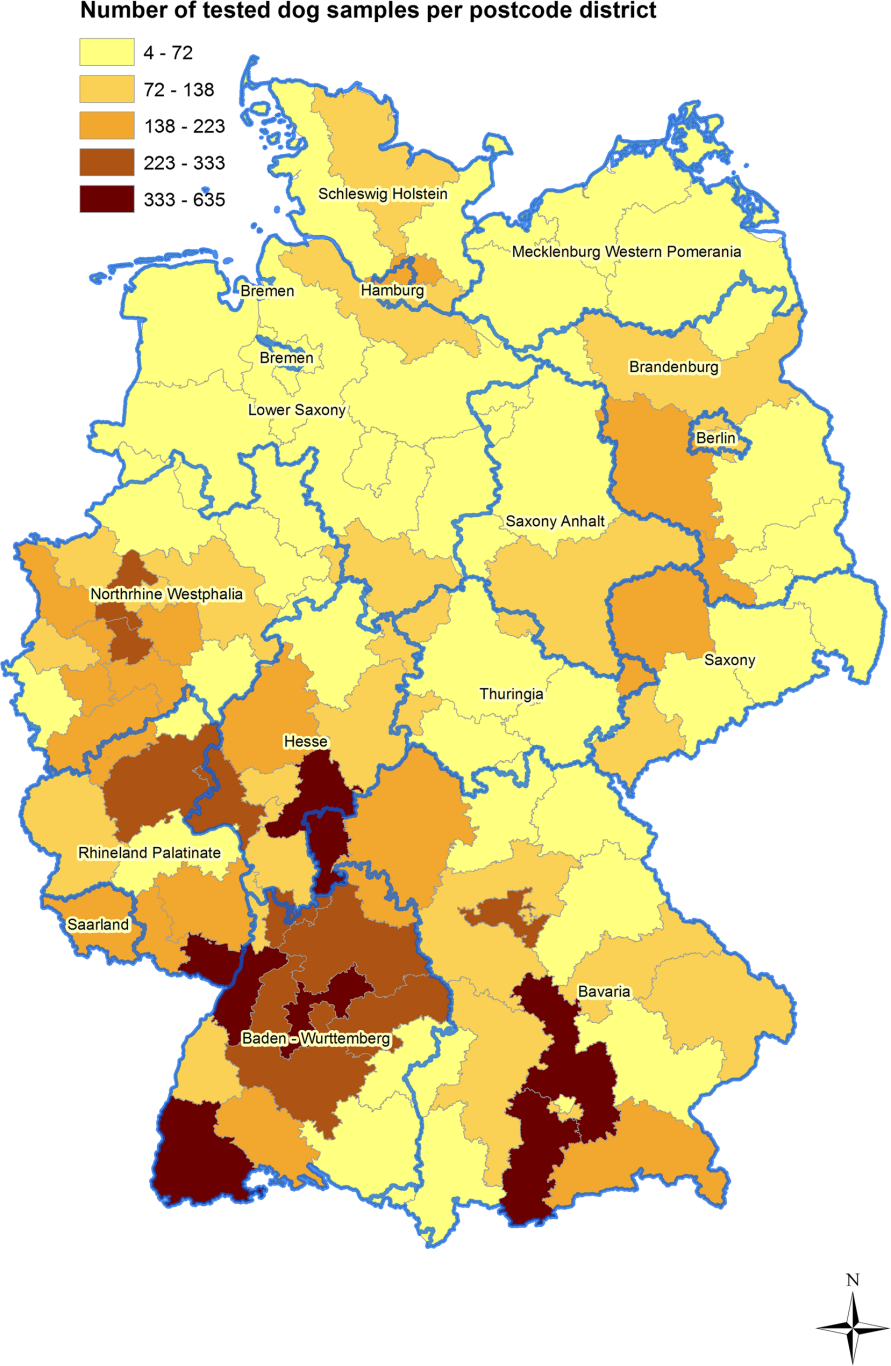
Number of dog faecal samples per postcode district

In total, samples were obtained from 1,745 postcode areas representing all 96 postcode districts in Germany. The number of samples per postcode district varied between 4 and 435 (Fig. 1). Most samples originated from Baden-Wuerttemberg (*n* = 3,387), followed by Bavaria (*n* = 2,512), North Rhine-Westphalia (*n* = 2,251) and Hesse (*n* = 1,400) (Additional file 1: Figure S1). In general, the number of faecal samples tested by Baermann funnel technique increased considerably over the years (Additional file 1: Figure S1).

**Fig. 2:**
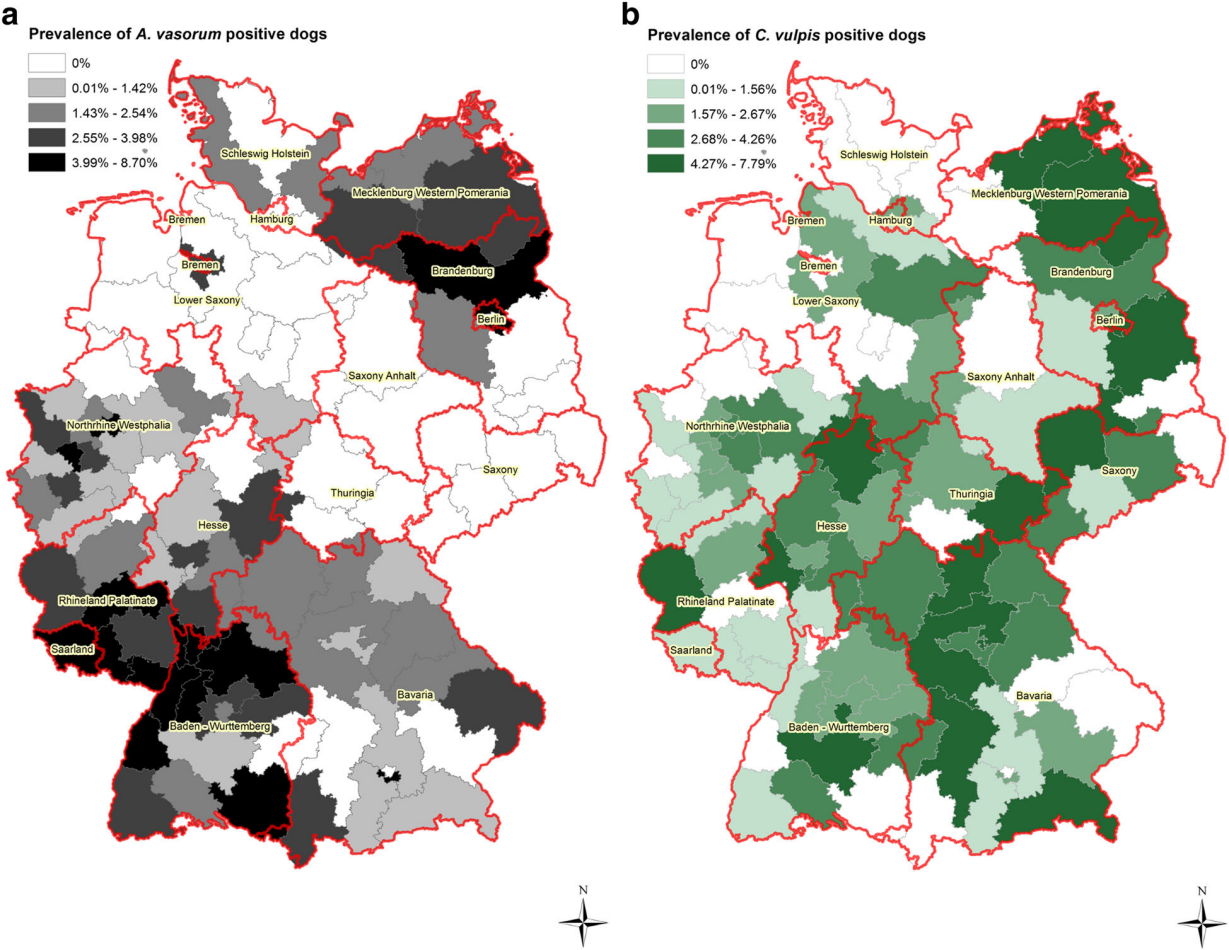
**a** Prevalence of canine *Angiostrongylus vasorum* infections per postcode district in 2003–2015 in Germany. **b** Prevalence of canine *Crenosoma vulpis* infections per postcode district in 2003–2015 in Germany

The diagnosed prevalence of *A. vasorum*-infected dogs varied among the German federal states between 0.01 and 8.7%. The highest proportion of *A. vasorum*-positive dogs was recorded in postcode districts of Baden-Wuerttemberg, Rhineland-Palatinate, Saarland, North Rhine-Westphalia, Berlin and Brandenburg. Here, the numbers of positive animals per postcode district ranged between 4.0 and 8.7% (Fig. 2a). The lowest proportion of *A. vasorum*-positive samples was detected in postcode districts from Schleswig-Holstein and Thuringia with 0.01–2.54% (Fig. 2a). In the case of *C. vulpis*, most postcode districts with a high prevalence (4.27–7.79%) were in the eastern parts of Germany (Fig. 2b). The lowest number of *C. vulpis*-positive dogs was observed in postcode areas from Saxony-Anhalt, Schleswig-Holstein and Lower Saxony (0.01–2.67%, 0.01–4.26% and 0.01–2.67%, respectively). The mean prevalence considering the entire study period (13 years) and the total study area was 2.27% (95% CI: 2.02–2.50) for *A. vasorum*-positive dogs and 2.25% (95% CI: 2.00–2.52) for *C. vulpis* infections.

**Fig. 3:**
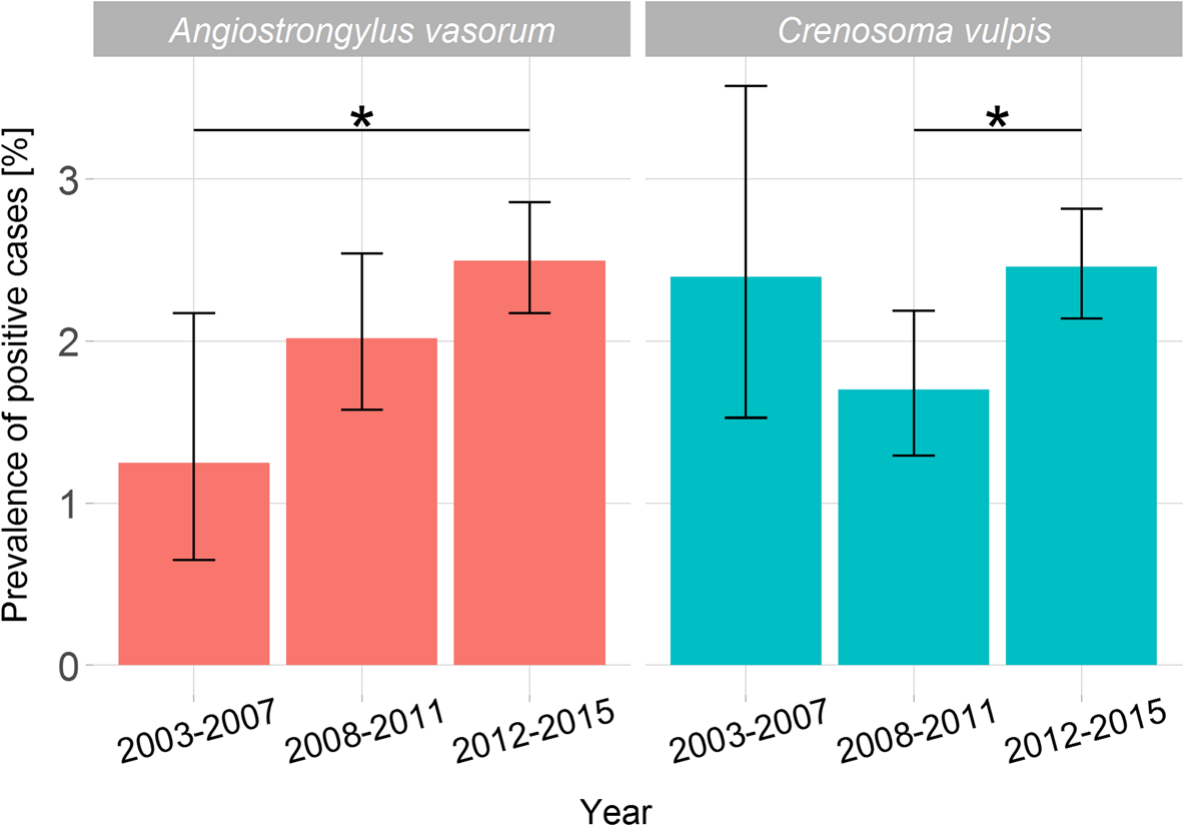
Percentage of *Angiostrongylus vasorum*- and *Crenosoma vulpis*-positive cases per defined time spans. Statistically significant differences are marked by horizontal bars and asterisks

Since the annual sample size in the first seven years was markedly lower than in the last six years of the study (2010–2015), the data were grouped into three time spans 2003–2007, 2008–2011 and 2012–2015 (Fig. 3). Considering these periods, the percentage of *A. vasorum*-positive cases increased statistically significantly between the first and the third-time span (pairwise Fisher’s exact test, holm corrected *P*-value < 0.05) (Fig. 3). However, no significant differences were recorded between the first and the second as well as between the second and third-time span for the *A. vasorum* prevalence (Fig. 3). After not significantly decreased *C. vulpis* prevalences had been found between the first and second time span, the prevalences increased statistically significantly between the second and the third-time span (pairwise Fisher’s exact test, Holm’s corrected *P*-value < 0.05) (Fig. 3).

Coinfections of *A. vasorum* and *C. vulpis* were also found in 10 animals. There was neither spatial nor temporal correlation between these infected animals (Additional file 2: Figure S2).

### Spatial differences in the occurrence of canine *A. vasorum* and *C. vulpis* infections

Spatial differences in the occurrence of *A. vasorum* and *C. vulpis* infections were observed in Germany. A statistically significantly higher proportion of *A. vasorum*-positive dogs compared to *C. vulpis*-infected ones was recorded in Baden-Wurttemberg and Rhineland Palatinate (summarised data from respective postcodes; Fisher’s exact test: *P*-value < 0.05) (Table 1). In contrast, a significantly higher proportion of *C. vulpis* compared to *A. vasorum* infections was detected in Bavaria, Hesse and Saxony (Table 1).

**Table 1:** *Angiostrongylus vasorum*- and *Crenosoma vulpis*-infected dogs in the federal states of Germany

Federal state	Totals	*A. vasorum-*positive	*C. vulpis-*positive	*P*-value	95% CI lower	95% CI upper
**Baden-Wuerttemberg**	**3,387**	**121**	**70**	**< 0.0001**	**1.292**	**2.401**
***Bavaria***	***2,512***	***35***	***64***	***0.004***	***0.346***	***0.832***
Berlin	389	14	14	1	0.435	2.298
Brandenburg	273	6	6	1	0.264	3.793
Bremen	28	1	0	1	0.026	Inf
Hamburg	159	0	3	0.248	0	2.411
***Hesse***	***1,400***	***25***	***46***	***0.016***	***0.313***	***0.895***
Lower Saxony	467	1	7	0.069	0.003	1.106
Mecklenburg Western Pomerania	116	2	5	0.446	0.037	2.449
North Rhine-Westphalia	2,251	46	39	0.511	0.752	1.87
**Rhineland-Palatinate**	**745**	**29**	**14**	**0.029**	**1.071**	**4.367**
Saarland	110	5	0	0.06	0.933	Inf
***Saxony***	***307***	***0***	***10***	***0.002***	***0***	***0.437***
Saxony-Anhalt	134	0	1	1	0	39.001
Schleswig-Holstein	283	2	1	1	0.104	118.746
Thuringia	121	1	5	0.213	0.004	1.775

*Note*: Text in bold: statistically significantly increased prevalence of *A. vasorum*-positive dogs in the respective federal state. Text in bold italic: statistically significantly increased prevalence of *C. vulpis*-positive dogs in the respective federal state

### *A. vasorum-* and *C. vulpis*-positive time period per postcode district

Each German postcode district was sampled on average for 11 years. When the respective postcode districts revealed at least one *A. vasorum* and/or *C. vulpis* positive sample within a year, this year was considered as positive for the corresponding infection.

**Fig. 4:**
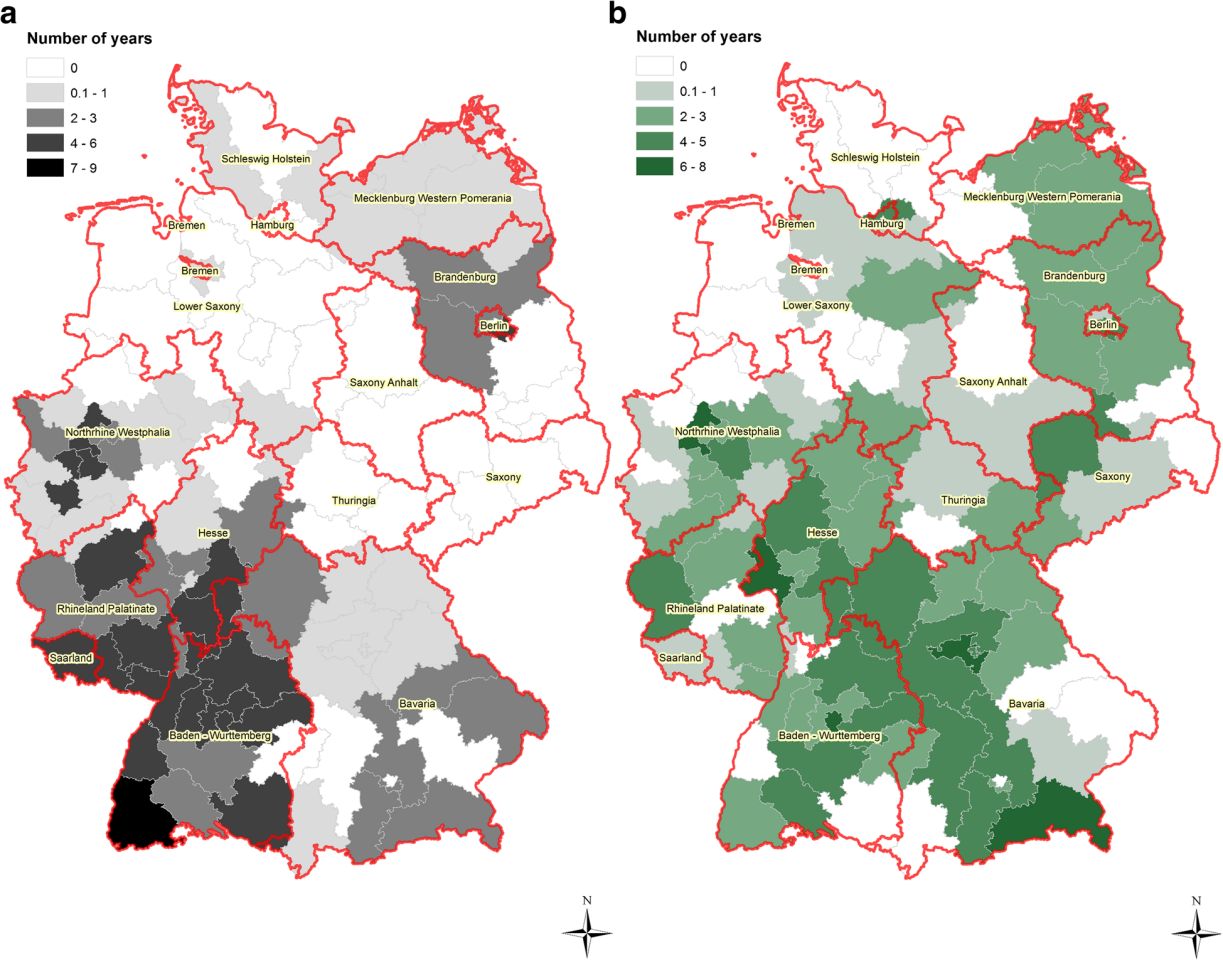
**a** Number of *Angiostrongylus vasorum*-positive years per postcode district. If a postcode district contained at least one positive sample within one year, the year and district was regarded as positive. **b** Number of *Crenosoma vulpis*-positive years per postcode district. If a postcode district contained at least one positive sample within one year, the year and district was regarded as positive

In 22 of 63 *A. vasorum*-positive postcode districts, the infection was detected in one out of 13 years (Fig. 4a). In the remaining *A. vasorum*-positive postcode districts the infection was recorded during two to nine years (Fig. 4a).

In 17 of 69 *C. vulpis*-positive postcode districts, *C. vulpis* infection was found in one out of 13 years (Fig. 4b). In the remaining postcode districts the number of *C. vulpis*-positive years varied between two and eight years (Fig. 4b). A high number of *A. vasorum*-positive years (4–9) was recorded for postcode districts of the Federal States of Baden-Wuerttemberg, Rhineland-Palatinate, Hesse, North Rhine-Westphalia and Berlin (Fig. 4a). On the federal state level, *C. vulpis*-positive areas were largely overlapping with those of high *A. vasorum* prevalence, i.e. 4–8 *C. vulpis*-positive years per postcode district were observed in Baden–Wuerttemberg, Hesse, Bavaria, Saxony, North Rhine-Westphalia and Berlin (Fig. 4b).

### Influence of season and age on canine *A. vasorum* and *C. vulpis* prevalences

**Fig. 5:**
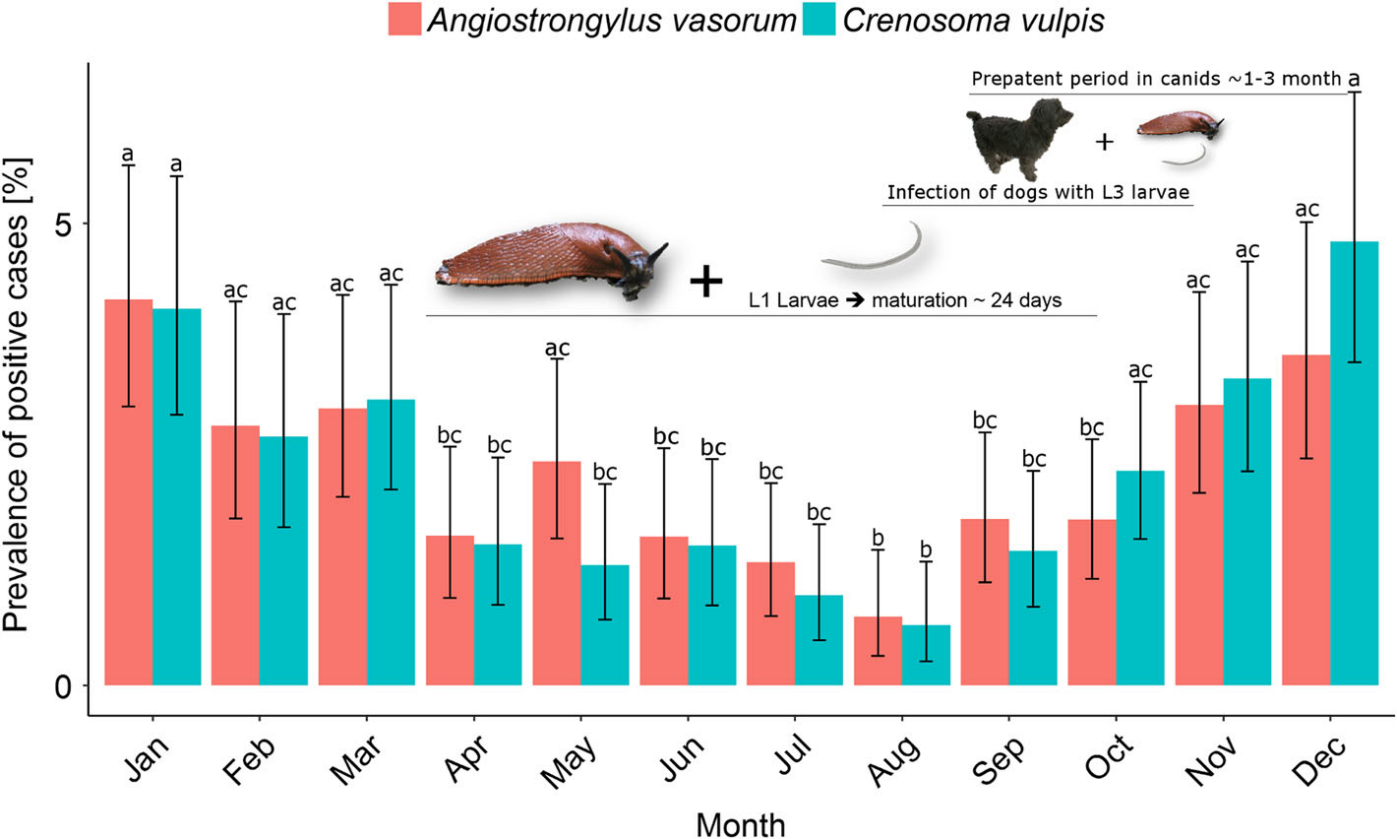
Seasonality of *Angiostrongylus vasorum* and *Crenosoma vulpis* infections. Each bar represents the mean monthly prevalence (calculated for 2003–2015) of *A. vasorum* (*red bars*) and *C. vulpis* (*blue bars*). Whiskers show 95% confidence intervals. Different letters above the whiskers indicate statistically significant differences between the proportions of positive and negative results (pairwise Fisher’s test). The horizontal bars represent stages of the life-cycle refer to a hypothesis to explain the seasonal pattern of diagnosed *A. vasorum* and *C. vulpis* infections in domestic dogs

Considering the proportions of parasite-positive samples on a monthly basis over the year, seasonal effects on larval shedding were observed for both parasites. The highest proportions of *A. vasorum-* and *C. vulpis*-positive samples were recorded during the winter months December and January. The lowest numbers of larval-shedding animals were diagnosed in April to September. These results indicate that most animals shed larvae throughout the winter season (Fig. 5). The prevalence in August was statistically significantly lower than that in November, December, January, February and March for both lungworms (Fig. 5; pairwise Fisher’s exact test: Holm’s corrected *P*-value < 0.05). Furthermore, the proportion of both *A. vasorum-* and *C. vulpis*-positive animals was significantly higher in January than in April, June, July, August and September (Fig. 5).

Dogs older than 12 months were statistically significantly less frequently infected with *A. vasorum* and *C. vulpis* than younger animals (Fisher’s test *P*-value < 0.001). Thus, *A. vasorum* and *C. vulpis* larval shedding were negatively associated with the age of dogs [Univariable generalised linear model: Estimate: -0.1165; Pr(>|z|) = 0.001 for *A. vasorum* infections and Estimate: -0.0809; Pr(>|z|) = 0.0273 for *C. vulpis* infections].

### Association of a digital landscape model of Germany with *A. vasorum* and *C. vulpis* prevalences in tested dog population

#### Univariable analysis

Univariable statistical analysis revealed statistically significant associations of five out of 15 tested independent variables with diagnosed *A. vasorum* infection. The landscape factors woody plant area, broadleaf forest and mixed forest were identified as risk factors, while higher proportions of softwood forest or agricultural fields turned out as protective factors (Additional file 3: Table S1).

For diagnosed *C. vulpis* infections, statistically significant associations were recorded for four out of 15 independent variables (Additional file 3: Table S1). Higher proportions of moorland or housing areas were risk factors for diagnosed *C. vulpis* infections, whereas agricultural fields and water bodies were protective (Additional file 3: Table S1).

#### Multivariable analysis

Independent variables showing *P*-values ≤ 0.2 were included in a full logistic regression model for multivariable analysis [26]. Thus, the full model for *A. vasorum* included the following variables: age of the dogs, *A. vasorum* and *C. vulpis* seasonality (mean prevalence per month), agricultural field, other agriculture areas, woody plant area, moorland, broadleaf forest, softwood forest, mixed forest.

The full model revealed statistically significant associations of diagnosed *A. vasorum* infections with woody plant area, mixed forest and *A. vasorum* seasonality as risk factors as well as age as a protective factor with a pseudo-*R* ^2^ value of 0.05 (Additional file 4: Table S2). The highest significant association was found with *A. vasorum* seasonality followed by woody plant area (Additional file 4: Table S2). After stepwise selection of variables from the tested model, the most parsimonious model was achieved with following variables: other agriculture areas (risk factor), woody plant area (risk factor), moorland (protective factor), broadleaf forest (risk factor), mixed forest (risk factor), age (protective factor) and *A. vasorum* seasonality (risk factor) (Table 2). After stepwise elimination of variables, the final model showed a pseudo-*R* ^2^ value of 0.055 (Table 2). Similar to the full model, the strongest association in the final model was recorded for *A. vasorum* seasonality followed by woody plant area (Multivariable generalized linear model: Estimate: 46.66; Pr(>|z|) < 0.0001 for seasonality and Estimate: 11.49; Pr(>|z|) = 0.0003 for woody plant area) (Table 3). An empirical ROC analysis revealed the same predictive values/accuracy (AUC = 0.7) for both, the full and the final model.

**Table 2:** Results of the final multivariable logistic regression models for *Angiostrongylus vasorum* and *Crenosoma vulpis* after controlled stepwise elimination of variables

Variable	Estimate	SE	*z-*value	Pr(>|z|)	AIC	Pseudo-*R* ^2^	Parasite
Other agriculture	0.98	0.63	1.55	0.12	2,157.48	0.055	*A. vasorum*
**Woody plant area**	**11.49**	**3.18**	**3.61**	**0.0003**			
Moorland	-23.97	16.17	-1.48	0.13			
Broadleaf forest	1.54	1.02	1.51	0.13			
Softwood forest	-0.93	0.60	-1.53	0.12			
**Mixed forest**	**2.02**	**0.41**	**4.93**	**8.08e** ^**-07**^			
**Age in month**	**-0.12**	**0.03**	**-3.39**	**0.0007**			
***A. vasorum*** **prevalence per month**	**46.66**	**6.72**	**6.94**	**4.03e** ^**-12**^			
Bodies of water	-4.43	2.40	-1.85	0.06	2,132.82	0.034	*C. vulpis*
**Agricultural field**	**-1.02**	**0.39**	**-2.61**	**0.009**			
**Age in month**	**-0.07**	**0.04**	**-2.04**	**0.04**			
***C. vulpis*** **prevalence per month**	**38.06**	**5.18**	**7.35**	**1.99e** ^**-13**^			

*Note*: Statistically significant (*P* ≤ 0.05) associations are displayed in bold

*Abbreviations*: *SE* standard error, *AIC* Akaike’s Information Criterion

**Table 3:** Generalized linear mixed model using postcode as a random intercept

Variable	Estimate	SE	*z*-value	Pr(>|z|)	Residual deviance	AIC	Parasite
**Intercept**	**-5.49**	**0.37**	**-14.84**	**7.91e** ^**-50**^	2,111.68	2,131.68	*A. vasorum*
Other agriculture	0.70	0.80	0.87	0.38			
**Woody plant area**	**10.93**	**4.08**	**2.68**	**0.01**			
Moorland	-23.49	19.7	-1.19	0.23			
Broadleaf forest	2.06	1.24	1.66	0.1			
Softwood forest	-0.41	0.77	-0.53	0.59			
**Mixed forest**	**2.11**	**0.56**	**3.75**	**1.79e** ^**-04**^			
***A. vasorum*** **prevalence per month**	**46.79**	**6.93**	**6.75**	**1.45e** ^**-11**^			
Age in months	-0.12	0.04	-3.31	9.39e^-04^			
**Intercept**	**-4.3**	**0.29**	**-14.76**	**2.58e** ^**49**^	2,110.3	2,122.3	*C. vulpis*
Bodies of water	-4.12	2.58	-1.59	0.11			
Field	-1.14	0.44	-2.56	0.01			
Age in months	-0.08	0.04	-2.11	0.03			
*C. vulpis* prevalence per month	38.35	5.36	7.16	8.32e^-13^			

*Note*: Statistically significant (*P* ≤ 0.05) associations are displayed in bold

*Abbreviations*: *SE* standard error, *AIC* Akaike’s Information Criterion

In the full multivariable model for *C. vulpis*, age, *C. vulpis* seasonality, bodies of water, agricultural field, moorland, housing area, gender and seasonality were included as independent variables. Significant associations between *C. vulpis* positivity were found with the agricultural field (protective factor), seasonality (risk factor) and age (protective factor) (Table 2). The strongest association in this model was recorded for *C. vulpis* seasonality (multivariable generalised linear model: Estimate: 38.06; Pr(>|z|) < 0.0001) (Table 3). The lowest association (negative) was observed with agricultural field (Multivariable generalized linear model: Estimate: -1.02; Pr(>|z|) = 0.009) (Table 3). The most parsimonious model was achieved with following variables: bodies of water, agricultural field, age and *C. vulpis* seasonality (Table 3). The pseudo-*R* ^2^ value for this final model was 0.034. The empirical ROC analysis of the full and final models revealed AUC values of 0.66 for both models.

#### Generalised linear mixed model (GLMM) analysis

**Fig. 6:**
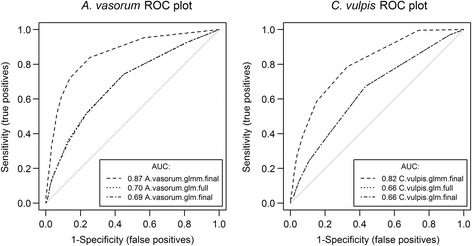
ROC analysis for the predictive performance of the models

Since animals originating from the same postcode area are more likely to live in similar landscapes, a generalised linear mixed model using the postcode area as a random intercept was applied. The results of these linear mixed models confirmed the significant association of the infection status regarding *A. vasorum* and *C. vulpis* in the final logistic regression models (Table 3). Introducing a random intercept for spatial information improved the models as demonstrated by markedly higher AUC values (AUC_*A. vasorum glmm*_ = 0.87); AUC_*C. vulpis glmm*_ = 0.82) in the empirical ROC analysis (Fig. 6).

## Discussion

*Angiostrongylus vasorum* and *C. vulpis* infections of domestic dogs may lead to severe dyspnoea or, in the case of angiostrongylosis, even to fatal coagulopathies or pulmonary hypertension [27]. Canine angiostrongylosis also induces a wide range of other clinical signs, such as neurological symptoms, intracranial haemorrhage and gastrointestinal bleeding [1]. *Angiostrongylus vasorum* infections are not only life-threating but also have a considerable economic impact on the dog owners. Consequently, an increased knowledge on the epidemiology of these lungworms is necessary to better understand and control the risk of infection. We, therefore, analysed 12,682 samples and epidemiological data from *A. vasorum-* or *C. vulpis*-infected German dogs obtained between 2003 and 2015 and calculated risk factors as well as temporal and spatial differences of *A. vasorum* and *C. vulpis* infections. It is important to note that the samples were obtained by convenience sampling and were likely to be biased in the sense that they originated from dogs suspicious for lungworm infections. Furthermore, we observed a spatial sampling bias, i.e. markedly higher numbers of samples were obtained from postcode regions with a higher urbanisation level. The sampled population may, therefore, be biased, and the estimated *A. vasorum* and *C. vulpis* prevalences might not be representative of the total dog population in Germany. Nevertheless, the current data may give new insights into the spatial and temporal dynamics of *A. vasorum* and *C. vulpis* infections in dog populations exhibiting clinical signs.

An expansion of *A. vasorum* endemic areas and/or the emergence of this parasite in regions previously believed to be free was recently reported for Great Britain, Canada, the Netherlands or regions of Scandinavia [2, 28]. Moreover, Taylor et al. [2] reported an increasing *A. vasorum* prevalence in British foxes. The current analyses also showed that the prevalence of *A. vasorum* and *C. vulpis* infections increased from 2003 until 2015 and from 2008 until 2015, respectively, which might at least in part be linked to an increasing awareness among veterinarians and dog owners, leading to higher numbers of dogs being tested for these infections as also reported for the mainland of the United Kingdom [29]. This is in accordance with the fact that the yearly sample numbers of the current study increased considerably from 2003 to 2015 (Additional file 1: Figure S1). Overall, prevalences of 2.28 and 2.25% were detected for canine *A. vasorum* and *C. vulpis* infections, respectively, using the Baermann funnel method. The advantage of this technique is its simplicity and ability to provide a result for different nematode larvae once the samples have been collected. Nonetheless, in contrast to ELISA methods [30], this technique fails to detect prepatent infections and requires three consecutive days faecal sample collection for sufficient sensitivity. With the Baermann funnel test, the larval burden in an animal cannot be reliably determined, but rather the presence or absence of a lungworm infection. Moreover, only experienced analysts are required to differentiate between *A. vasorum* and *C. vulpis* larvae. It also has to be considered that only one faecal sample was examined per animal in the current study. Given that the shedding of *A. vasorum* and *C. vulpis* larvae can be intermittent [1, 5], infections may have been missed. Thus, it appears likely that we underestimated the prevalences of both nematodes.

Chapman and colleagues found 0.67% (23 of 3,407) *A. vasorum*-positive cases in the United Kindom for the period 1999–2002; most of the cases (22/23) originated from the south-east of England [31]. In Italy, Traversa and colleagues found 2.3% *A. vasorum*-positive cases in dogs with clinical signs compatible with canine angiostrongylosis [32]. In Belgium, Canonne et al. [33] detected 3% *A. vasorum*-positive dogs in 225 registered canine cases with respiratory disease. In Switzerland, 3.08% of 6,136 tested dogs had antibodies against *A. vasorum*-antigen and in 2.17% of this group circulating antigen was detected by ELISA [18]. Studies reporting *C. vulpis* infections in domestic dogs in different countries also showed different proportions of positive animals [3, 7, 34–36]. As the study designs differed considerably (e.g. sample size, spatial and temporal sampling, kind of sample preselection, test methods, etc.), it is difficult to compare the prevalence determined in the present study with those reported for other European countries. This indicates that harmonised epidemiological studies are required to compare *A. vasorum* and *C. vulpis* prevalences in dog populations among different areas in Europe.

Although the samples revealed a spatial sampling bias with the highest number of faecal samples originating from southwestern Germany, we still observed significant spatial differences in the occurrence of *A. vasorum* and *C. vulpis* infections. While the highest *A. vasorum* prevalences were observed in southwestern postcode districts, the highest proportions of *C. vulpis*-infected animals were recorded in eastern/southeastern Germany. So far, the basis of these geographical differences remained unclear. Both parasites use slugs and snails as intermediate hosts and foxes serve for both nematodes as a reservoir host. However, the actual spectrum of intermediate hosts may influence the epidemiology of these parasites as previously shown for *C. vulpis* [37]. Spatial spreading of *A. vasorum* and *C. vulpis* may also be influenced by movements of wild foxes (e.g. dispersal of juvenile animals), dogs (e.g. travelling) and intermediate hosts as already discussed by others [13, 14]. However, further studies are needed to understand the contribution of environmental factors as well as different species of intermediate and final hosts to the epidemiology of lungworm infections.

The spatial clusters of *A. vasorum* cases in southwestern Germany corresponded well to previously reported endemic areas of this parasite [11, 38]. Our recent data confirm rather high prevalences of *A. vasorum* in red foxes in these areas (Rhineland-Palatinate: 27.3%) [39]. Hartwig et al. [4] reported *A. vasorum* infections in foxes in the federal state of Brandenburg, suggesting this parasite to be endemic in the northeastern part of Germany. Similarly, our data also showed relatively high proportions of *A. vasorum*-infected dogs in the North-East of Germany (Brandenburg and Berlin) potentially indicating a northeastern expansion of the parasite from the historically known endemic areas in the southwestern parts of Germany. However, this trend should be handled with care as it is unclear whether the suspected new endemic areas result from real spreading or from enhanced awareness and improved diagnosis in areas previously deemed free. We also found a considerable number of canine *A. vasorum* infections in central parts of Germany (Hesse). This area corresponds to a region with a high *A. vasorum* prevalence in red foxes (19.1%) [39] emphasising the role of the red fox as a reservoir host in the epidemiology of this nematode.

One further possible explanation for described the spread of *A. vasorum* infections beyond the known endemic foci may be the influence of climatic factors, which may contribute to the survival of the larvae of these nematodes and their intermediate host [40]. However, this hypothesis must be tested in future studies.

We further analysed for how many years postcode districts remained positive for canine *A. vasorum* and *C. vulpis* infections. The longest duration of *A. vasorum*-positivity was recorded for West and South-West Germany, i.e. for areas, which are well-known as endemic for this parasite. In Berlin, *A. vasorum*-positive cases were recorded for 7–9 years while Brandenburg and Bavaria were positive for only 2–3 years. These findings may support the emergence of new endemic regions for *A. vasorum* infections in domestic dogs in Germany. However, this observation needs to be confirmed by future nationwide epidemiological studies.

At least over two years, *C. vulpis* cases were recorded in 12 of 16 federal states in Germany, which clearly confirms that *C. vulpis* is endemic in most parts of Germany [3, 11].

For both parasites, a clear seasonal influence on canine prevalences was observed with the highest levels throughout the winter period (December-February) followed by a decline until July/August. Morgan et al. [17] discussed an increased population of gastropods in autumn causing a high abundance of third larval stages as a reason for this seasonal trend. Our observations support the view that large gastropod populations exist in spring and late summer/early autumn in Germany, while in early summer or winter the slug abundance is reduced. This may facilitate gastropod infections during these time spans. In accordance, Ferdushy et al. [41] found third stage larvae of *A. vasorum* in naturally-infected gastropods in September-October. Own yet unpublished data on slug infections revealed that the highest proportion of German slugs were infected with *A. vasorum*-L3 in summer and autumn (9.1 and 4.7%, respectively, M. Lange, personal communication). However, decreasing canine prevalences from winter to autumn may also be the result of prophylactic or therapeutic deworming activities, since Morgan et al. [14] reported that deworming measures being applied 12 weeks or less before the infection has a potential protective effect.

It is accepted that young dogs are more often infected with *A. vasorum* than older ones [14, 31]. Thus, the young, untreated dog population (≤12 months) may also have contributed to the observed seasonal trends. Chapman and colleagues [31] reported that the median age of clinically affected dogs was ten months. Similarly, Koch & Willesen [1] found that more than 50% of infected dogs were younger than 12 months and Barutzki & Scharper [11] identified 1–2 years-old dogs as the main *A. vasorum*-infected subpopulation in Germany. Accordingly, our data confirm that young dogs (≤ 12 months) were significantly more often *A. vasorum*-infected than older dogs within the population suspected for lungworm infections. Additionally, logistic regression analysis of the present data revealed that the age had a protective effect, i.e. older dogs were significantly less likely to be infected with *A. vasorum* or *C. vulpis*. This effect may be linked to acquired immunity. Similar results were reported by Morgan et al. [14] for *A. vasorum* infections.

Several risk factors for *A. vasorum* infections have already been described. Lurati et al. [18] showed that the altitude (≥700 m above sea level) plays a role as a limiting factor for *A. vasorum* transmission in Switzerland. For southern parts of Great Britain, Morgan et al. [14] reported on the factors age (protective), season (higher risk of *A. vasorum* infection in winter) and deworming history (protective) as factors influencing the probability of *A. vasorum* infections. So far, no data are available on the impact of geographical factors, such as landscape elements or land-use, as risk or protective factors for canine *A. vasorum* or *C. vulpis* infections. We identified woody plant area, broadleaf forest, mixed forest and seasonality as risk factors for canine *A. vasorum* infections in Germany by univariable logistic regression analysis, while agricultural field represented a protective factor. Woody plant area and broadleaf forest revealed the strongest positive association with *A. vasorum* infections. Dog walking areas in German cities are often located in parks or recreational areas in the surroundings of the cities. They include forested and woody plant area areas, which represent suitable habitats for gastropods and for synanthropic foxes.

Interestingly, univariable logistic regression revealed some overlapping risk and protective factors for canine *A. vasorum* and *C. vulpis* infections but also identified differences. For both parasites, age, seasonality and agricultural field showed-up as protective factors. However, moorland and housing area were additional risk factors for *C. vulpis* infections, while woody plant area, broadleaf forest, mixed forest (risk factors for *A. vasorum*) did not apply for this parasite. These differences may point at different habitat requirements for both parasites. While sharing several steps in their life-cycles, they may prefer different intermediate host spectra or habitat conditions. In mixed forest and groove, *Arion lusitanicus* and *Limax maximus* slugs can be expected, whereas *A. lusitanicus* and *Deroceras reticulatum* can be mainly found in areas of crop production, i.e. agriculturally used fields (http://www.molluscs.at/gastropoda/terrestrial/helix.html?/gastropoda/terrestrial/helix/main.html). There are several other slug and snail species, which use the above-mentioned habitats and represent potential intermediate hosts for *A. vasorum* and *C. vulpis*. Aziz and colleagues [12] found that larger slugs (e.g. *Arion* spp.) were frequently infected. Unfortunately, we could not find any epidemiological information about the occurrence of *C. vulpis* infections and only limited about *A. vasorum* infections in the intermediate hosts in Europe. Further representative studies on *C. vulpis* and *A. vasorum* prevalences in intermediate hosts from different landscape sites in Germany are required to improve our understanding of the epidemiology of these parasites.

The present study shows divergent geographical distributions and some divergent risk factors for *A. vasorum* and *C. vulpis* infections. Since foxes are known as reservoir wildlife hosts for both nematode species [2, 4, 42], it is tempting to speculate that these differences concern the respective intermediate hosts.

Since the sample size in the tested dog population was not representative of each postal code area or federal state, we cannot exclude that further environmental factors may be identified as protective or as risk variables in studies with an improved, i.e. representative, design.

Several studies reported that foxes represent the main reservoir of *A. vasorum*, as the prevalence in foxes seems to correlate with that in dogs [2, 15]. Furthermore, forest and other shelter-offering habitats represent typical breeding areas of foxes according to Keuling and colleagues [43]. Since no obvious genetic segregation of *A. vasorum* was found in dogs, foxes and coyotes [16], the transmission of different genetic clades of *A. vasorum* are most likely to occur between wild and domestic canids. Thus, *A. vasorum*-associated landscape risk factors (mixed forests and woody plant area) may also apply for fox infections.

## Conclusions

The data of the current study clearly demonstrate spatial differences in the occurrence of *A. vasorum* and *C. vulpis* infections of dogs in Germany*.* Risk factor analyses revealed overlapping, but also diverging aspects of *A. vasorum* and *C. vulpis* infections implicating differences in the biology of these parasites, presumably at the intermediate host level. The data also show a significant increase in the prevalence of *A. vasorum* and *C. vulpis* infections from 2010 to 2015 and a potential spread of *A. vasorum* to the northeastern part of Germany. Further studies that include the systematic sampling of dog populations, possibly also with a method for the assessment of the larval burden in the infected animals, are required to obtain representative prevalence estimates for *A. vasorum* and *C. vulpis* in the dog populations of different European countries. Such studies should also be complemented by research on intermediated hosts.

## Additional files

Additional file 1: Figure S1. Number of faecal samples tested in Baermann funnel method per year and federal state (TIFF 254 kb)
Additional file 2: Figure S2. Map presenting DLM factors defined by the final model together with *A. vasorum-* and *C. vulpis* - positive cases. (TIFF 12806 kb)
Additional file 3: Table S1. Univariable statistical analysis of associations several independent variables and infection with *A. vasorum* or *C. vulpis. (DOCX 16 kb)*

Additional file 4: Table S2. Results of the full multivariable logistic regression model for *A. vasorum* and *C. vulpis. (DOCX 14 kb)*

Additional file 5: Database 1. The data supporting the findings of present study. (CSV 2996 kb)

## Abbreviations

AIC: Akaike’s information criterion
ATKIS: Amtliches topographisch-kartographisches Informationssystem, Bundesamt für Kartographie und Geodäsie, Frankfurt, Germany; http://www.geodatenzentrum.de
AUC: Area under curve
GIS: Geographical information system
GLMM: Generalised linear mixed model analysis
ROC: Receiver operating characteristics
