# Supplementary figures and images for: GIS-supported epidemiological analysis on canine Angiostrongylus vasorum and Crenosoma vulpis infections in Germany
Source: Parasit Vectors. 2017 Feb 28;10:108. doi: 10.1186/s13071-017-2054-3 (PMC5330135; doi:10.1186/s13071-017-2054-3)

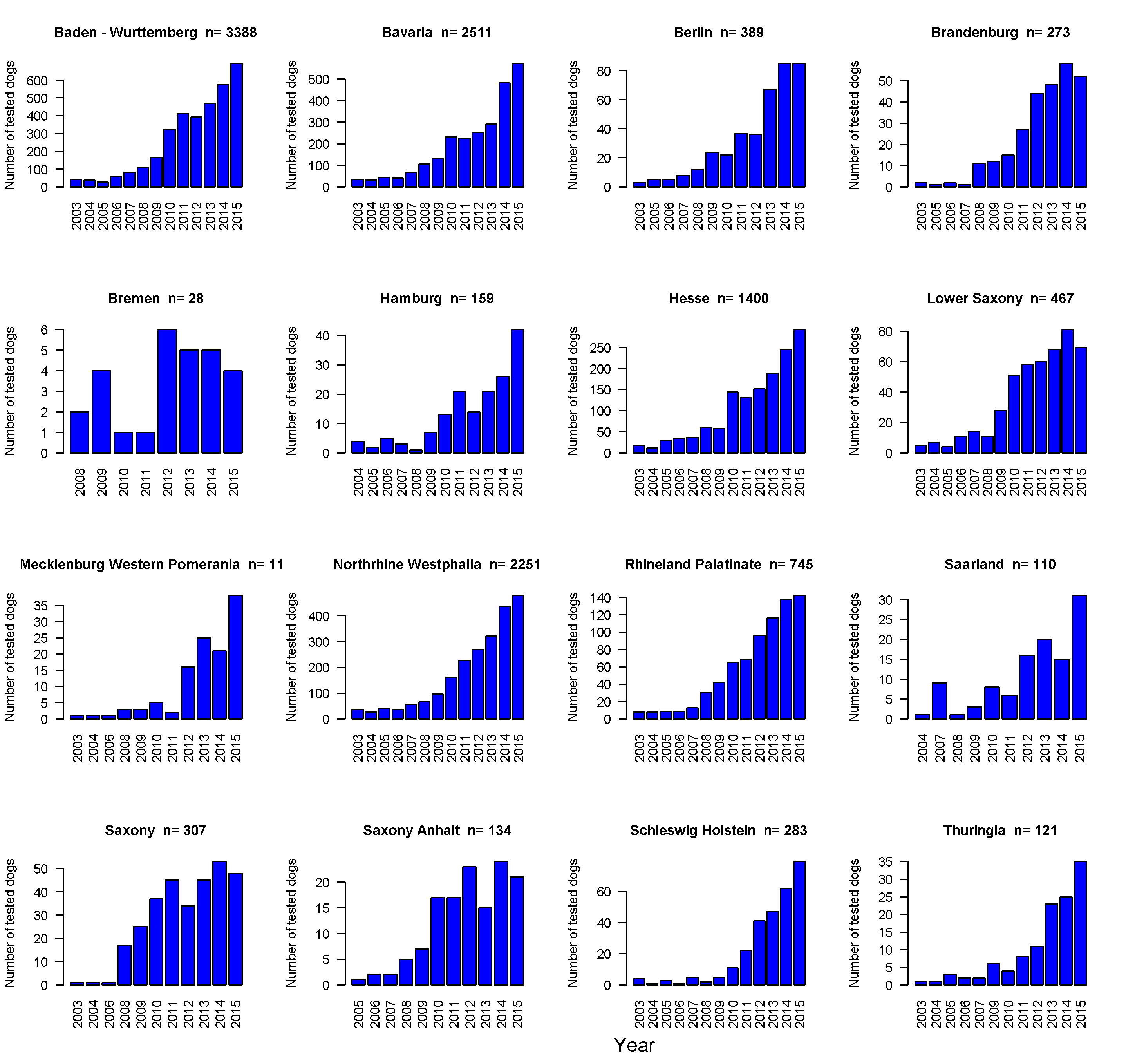

Supplement: Additional file 1: Figure S1. — Number of faecal samples tested in Baermann funnel method per year and federal state (TIFF 254 kb) [file 13071_2017_2054_MOESM1_ESM.tiff]

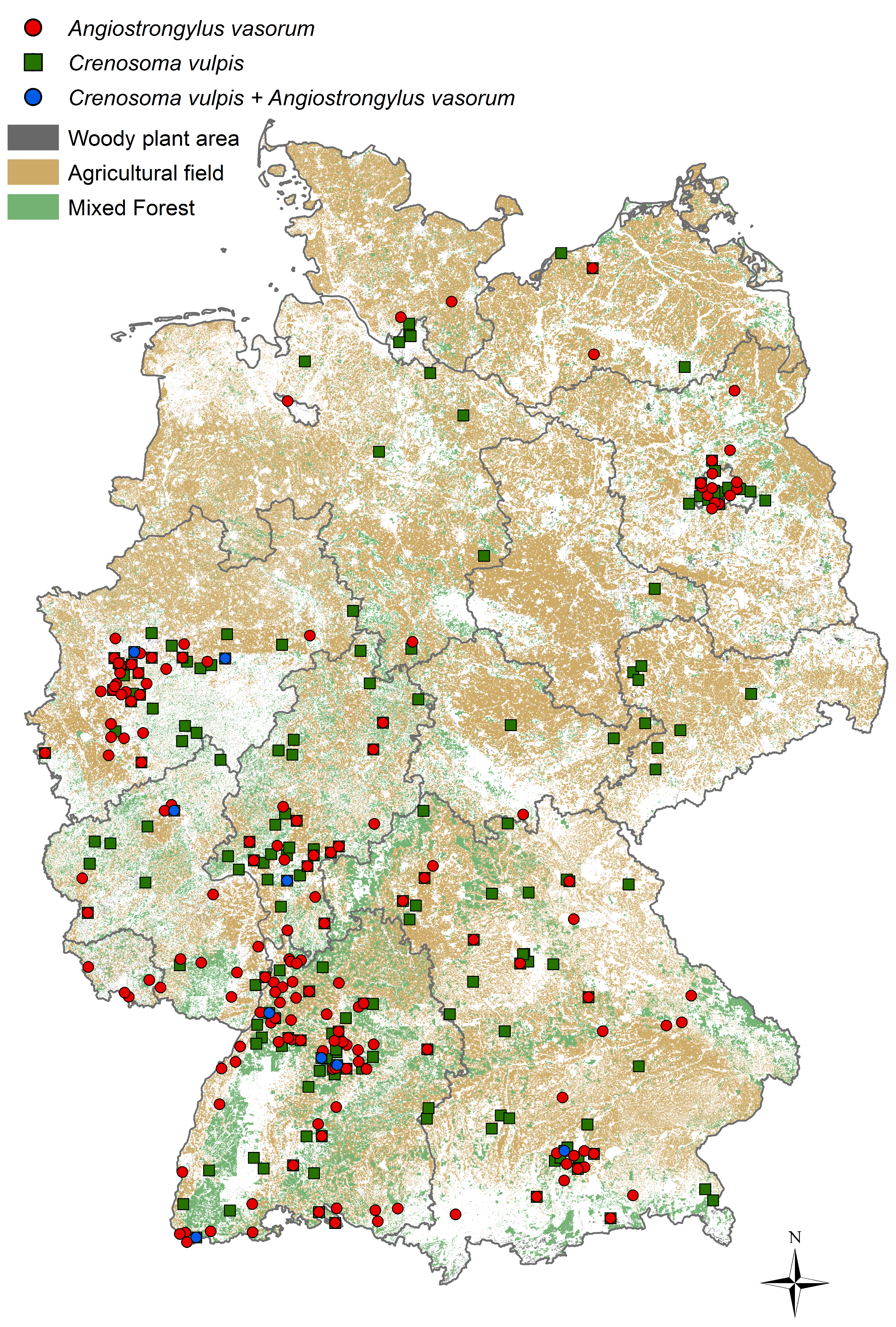

Supplement: Additional file 2: Figure S2. — Map presenting DLM factors defined by the final model together with A. vasorum- and C. vulpis - positive cases. (TIFF 12806 kb) [file 13071_2017_2054_MOESM2_ESM.tiff]
