# Supplementary material for: GIS-supported epidemiological analysis on canine Angiostrongylus vasorum and Crenosoma vulpis infections in Germany
Source: Parasit Vectors. 2017 Feb 28;10:108. doi: 10.1186/s13071-017-2054-3 (PMC5330135; doi:10.1186/s13071-017-2054-3)
Supplement: Additional file 3: Table S1. — Univariable statistical analysis of associations several independent variables and infection with A. vasorum or C. vulpis. (DOCX 16 kb) [file 13071_2017_2054_MOESM3_ESM.docx]

Additional file 3. Univariable statistical analysis of associations several independent variables and infection with A. vasorum or C. vulpis

| **Variable** | **Estimate** | **S.E.** | **z value** | **Pr(>\|z\|)** | **Null deviance** | **Residual deviance** | **AIC** | **Parasite** |
| --- | --- | --- | --- | --- | --- | --- | --- | --- |
| Bodies of water | -0.775 | 1.544 | -0.502 | 0.616 | 2749.6 | 2749.285 | 2753.29 | *A. vasorum* |
| Traffic | 0.439 | 1.435 | 0.306 | 0.76 | 2749.6 | 2749.468 | 2753.47 | *A. vasorum* |
| Agricultural field | -0.813 | 0.344 | -2.361 | 0.018 | 2749.6 | 2743.75 | 2747.75 | *A. vasorum* |
| Other agriculture | 0.998 | 0.523 | 1.911 | 0.056 | 2749.6 | 2746.052 | 2750.05 | *A. vasorum* |
| Groove | 10.29 | 2.575 | 3.995 | 0 | 2749.6 | 2735.983 | 2739.98 | *A. vasorum* |
| Moorland | -26.176 | 16.782 | -1.56 | 0.119 | 2749.6 | 2745.538 | 2749.54 | *A. vasorum* |
| **Broadleaf forest** | **1.746** | **0.871** | **2.005** | **0.045** | **2749.6** | **2745.778** | **2749.78** | ***A. vasorum*** |
| **Softwood forest** | **-1.835** | **0.539** | **-3.402** | **0.001** | **2749.6** | **2735.105** | **2739.1** | ***A. vasorum*** |
| **Mixed forest** | **1.942** | **0.344** | **5.646** | **0** | **2749.6** | **2721.11** | **2725.11** | ***A. vasorum*** |
| Bog | -3.107 | 4.496 | -0.691 | 0.489 | 2749.6 | 2749.048 | 2753.05 | *A. vasorum* |
| Leisure/sport park area | -1.111 | 1.248 | -0.89 | 0.373 | 2749.6 | 2748.723 | 2752.72 | *A. vasorum* |
| Housing area | -0.182 | 0.446 | -0.407 | 0.684 | 2749.6 | 2749.389 | 2753.39 | *A. vasorum* |
| **Age in month** | **-0.116** | **0.036** | **-3.274** | **0.001** | **2250.8** | **2240.402** | **2244.4** | ***A. vasorum*** |
| ***A. vasorum* prevalence per month** | **42.889** | **6.061** | **7.076** | **0** | **2749.6** | **2699.91** | **2703.91** | ***A. vasorum*** |
| ***C. vulpis* prevalence per month** | **29.631** | **4.6** | **6.442** | **0** | **2749.6** | **2709.357** | **2713.36** | ***A. vasorum*** |
| **Bodies of water** | **-5.275** | **2.4** | **-2.198** | **0.028** | **2726.9** | **2720.333** | **2724.33** | ***C. vulpis*** |
| Traffic | -0.313 | 1.646 | -0.19 | 0.849 | 2726.9 | 2726.916 | 2730.92 | *C. vulpis* |
| **Agricultural field** | **-0.871** | **0.348** | **-2.505** | **0.012** | **2726.9** | **2720.389** | **2724.39** | ***C. vulpis*** |
| Other agriculture | -0.024 | 0.559 | -0.043 | 0.966 | 2726.9 | 2726.951 | 2730.95 | *C. vulpis* |
| Groove | -2.782 | 3.459 | -0.804 | 0.421 | 2726.9 | 2726.284 | 2730.28 | *C. vulpis* |
| **Moorland** | **12.6** | **4.489** | **2.807** | **0.005** | **2726.9** | **2721.33** | **2725.33** | ***C. vulpis*** |
| Broadleaf forest | 0.534 | 0.934 | 0.572 | 0.568 | 2726.9 | 2726.632 | 2730.63 | *C. vulpis* |
| Softwood forest | 0.421 | 0.367 | 1.145 | 0.252 | 2726.9 | 2725.702 | 2729.7 | *C. vulpis* |
| Mixed forest | 0.416 | 0.398 | 1.045 | 0.296 | 2726.9 | 2725.89 | 2729.89 | *C. vulpis* |
| Bog | -0.283 | 4.142 | -0.068 | 0.946 | 2726.9 | 2726.948 | 2730.95 | *C. vulpis* |
| Leisure/sport park area | 0.385 | 1.13 | 0.34 | 0.734 | 2726.9 | 2726.839 | 2730.84 | *C. vulpis* |
| Housing area | 0.764 | 0.402 | 1.901 | 0.057 | 2726.9 | 2723.54 | 2727.54 | *C. vulpis* |
| **Age in month** | **-0.081** | **0.037** | **-2.208** | **0.027** | **2190.7** | **2185.964** | **2189.96** | ***C. vulpis*** |
| ***A. vasorum* prevalence per month** | **50.098** | **6.11** | **8.199** | **0** | **2726.9** | **2659.794** | **2663.79** | ***C. vulpis*** |
| ***C. vulpis* prevalence per month** | **41.186** | **4.608** | **8.939** | **0** | **2726.9** | **2648.652** | **2652.65** | ***C. vulpis*** |

Statistically significant (p ≤ 0.05) associations are displayed **in bold.**

S.E.: Standard error; AIC: Akaike’s Information Criterion
