# Supplementary material for: GIS-supported epidemiological analysis on canine Angiostrongylus vasorum and Crenosoma vulpis infections in Germany
Source: Parasit Vectors. 2017 Feb 28;10:108. doi: 10.1186/s13071-017-2054-3 (PMC5330135; doi:10.1186/s13071-017-2054-3)
Supplement: Additional file 4: Table S2. — Results of the full multivariable logistic regression model for A. vasorum and C. vulpis. (DOCX 14 kb) [file 13071_2017_2054_MOESM4_ESM.docx]

Additional file 4: Results of the full multivariable logistic regression model for A. vasorum and C. vulpis

| **Variable** | **Estimate** | **S.E.** | ***z* value** | **Pr(>\|z\|)** | **AIC** | **Pseudo-*R*^2^** | **Parasite** |
| --- | --- | --- | --- | --- | --- | --- | --- |
| Agricultural field | -0.42 | 0.42 | -0.99 | 0.32 | 2160.47 | 0.055 | *A. vasorum* |
| Other agriculture | 0.99 | 0.63 | 1.58 | 0.12 |  |  | *A. vasorum* |
| **Woody plant area** | **10.78** | **3.26** | **3.31** | **0.0009** |  |  | ***A. vasorum*** |
| Moorland | -24.32 | 16.08 | -1.51 | 0.13 |  |  | *A. vasorum* |
| Broadleave forest | 1.53 | 1.02 | 1.51 | 0.13 |  |  | *A. vasorum* |
| Softwood forest | -1.04 | 0.61 | -1.69 | 0.09 |  |  | *A. vasorum* |
| **Mixed forest** | **1.90** | **0.42** | **4.47** | **7.72e-06** |  |  | ***A. vasorum*** |
| **Age** | **-0.12** | **0.04** | **-3.38** | **0.0007** |  |  | ***A. vasorum*** |
| ***A. vasorum* seasonality** | **45.48** | **15.74** | **2.89** | **0.004** |  |  | ***A. vasorum*** |
| *C. vulpis* seasonality | 0.97 | 12.22 | 0.08 | 0.94 |  |  | *A. vasorum* |
| Bodies of water | -4.12 | 2.39 | -1.72 | 0.09 | 2135.56 | 0.035 | *C. vulpis* |
| **Agricultural field** | **-0.85** | **0.44** | **-1.94** | **0.05** |  |  | ***C. vulpis*** |
| Moorland | 9.29 | 6.08 | 1.53 | 0.13 |  |  | *C. vulpis* |
| Housing area | 0.41 | 0.49 | 0.83 | 0.41 |  |  | *C. vulpis* |
| **Age** | **-0.08** | **0.04** | **-2.08** | **0.04** |  |  | ***C. vulpis*** |
| *A. vasorum* seasonality | 16.27 | 16.14 | 1.01 | 0.31 |  |  | *C. vulpis* |
| ***C. vulpis* seasonality** | **26.80** | **12.32** | **2.18** | **0.03** |  |  | ***C. vulpis*** |

Statistically significant (p ≤ 0.05) associations are displayed **in bold.**

S.E.: Standard error; AIC: Akaike’s Information Criterion
